# The new affordances in the home environment for motor development - infant scale (AHEMD-IS): Versions in English and Portuguese languages

**DOI:** 10.1590/bjpt-rbf.2014.0112

**Published:** 2015-09-01

**Authors:** Priscila M. Caçola, Carl Gabbard, Maria I. L. Montebelo, Denise C. C. Santos

**Affiliations:** 1 Developmental Motor Cognition Lab, Department of Kinesiology, The University of Texas at Arlington, Arlington, TX, USA; 2 Child Motor Development Lab, Department of Health and Kinesiology, Texas A&M University, College Station, TX, USA; 3 Faculdade de Ciências Exatas e da Natureza, Universidade Metodista de Piracicaba (UNIMEP), Piracicaba, SP, Brazil; 4 Programa de Pós-Graduação em Fisioterapia e em Ciências do Movimento Humano, Faculdade de Ciências da Saúde, Universidade Metodista de Piracicaba (UNIMEP), Piracicaba, SP, Brazil

**Keywords:** affordances, motor development, infants, home

## Abstract

The home environment has been established as a crucial factor for motor development, especially in infants. Exploring the home environment can have significant implications for intervention, as it is common practice in physical therapy to have professionals advise patients on home activities. Since 2010, our group has been working on the development of the Affordances in the Home Environment for Motor Development - Infant Scale (AHEMD-IS), a parental self-reporting instrument designed to assess the quality and quantity of factors (affordances) in the home environment. In Brazil, the instrument has been translated as "Affordances no Ambiente Domiciliar para o Desenvolvimento Motor - Escala Bebê", and it has been extensively used in several studies that address infant development. These studies in Brazil and other parts of the world highly recommended the need for a normative sample and standardized scoring system. A description of the study that addressed that need, along with the English version of the questionnaire and score sheets, was recently published in the well-known and respected journal *Physical Therapy*. Our intent with the present short communication is to notify Brazilian investigators and clinicians of this latest update so they can download the new instrument, as well as present the Brazilian (Portuguese) version of the AHEMD-IS along with its scoring system.

The home environment has been established as a crucial factor for motor development, especially in infants^1^. Exploring the home environment can have significant implications for intervention, as it is common practice in physical therapy to have professionals advise patients on home activities. In general, such recommendations involve how to utilize different aspects of home space, toys, stimulation, and activities that are part of an infant's everyday life^2^. Since 2010, our group has been working on the development of the Affordances in the Home Environment for Motor Development - Infant Scale (AHEMD-IS), a parental self-reporting instrument designed to assess the quality and quantity of factors (affordances) in the home environment. More specifically, the instrument addresses the dimensions of Physical Space, Variety of Stimulation, and Play Materials in the home that are conducive to enhancing motor development of infants aged 3 to 18 months^1^. Since its first publication in 2011 (as a descriptive tool with preliminary validity guidelines), the AHEMD-IS has gained in popularity as a clinical tool and as a research outcome measure^3^^-^ 8.

In Brazil, the instrument has been translated as *"Affordances no Ambiente Domiciliar para o Desenvolvimento Motor - Escala Bebê"*, and it has been extensively used in several studies that address infant development. For example, it was an important tool in a study exploring the role of biological and environmental factors in infant development, supporting the argument that environmental factors are associated with infant motor development^3^. Similarly, the AHEMD-IS has been recently added as a supporting tool in a major study designed to measure the effects of intrauterine growth restriction^4^. From other perspectives of infant development, a study has established that the availability of fine- and gross-motor toys in the home environment represents the real use of these opportunities (affordances) by infants^5^. Furthermore, it appears that all dimensions of the AHEMD-IS are highly influenced by socioeconomic status (SES)^6^with the exception of Variety of Stimulation^7^, and there are differences in home affordances when exploring two diverse areas of Brazil^8^.

With that said, studies in Brazil and other parts of the world that utilized the AHEMD-IS highly recommended the need for a normative sample and standardized scoring system. A description of the study that addressed that need, along with the English version of the questionnaire and score sheets, was recently published in the well-known and respected journal *Physical Therapy* 1. Our intent with the present short communication is to notify Brazilian investigators and clinicians of this latest update so they can download the new instrument, as well as present the Brazilian (Portuguese) version of the AHEMD-IS along with its scoring system. The whole process of further development and validation of the instrument included three phases: (1) use of expert opinion for content validity; (2) administration to a large Brazilian sample and testing of reliability, consistency, and floor and ceiling effects; and (3) re-testing of internal consistency and determination of interpretability for the scoring system.

As described in the *Physical Therapy* article^1^, Phase 1 (expert opinion of content) resulted in an average agreement of 95% across all criteria and a reduction of items (46-41). Phase 2 results showed ICC values of .99 for interrater and .95 for intrarater reliability, with Cronbach's alpha values ranging between .64 and .82 for the dimensions and a Total value of .82 (.78-.86) for internal consistency. Ceiling effects were found on three questions of the Inside Space dimension and three in Variety of Stimulation. These results demonstrated the need for reduction in total items (41 to 35) and combination of the Space sections. Results in Phase 3 revealed an internal consistency of .77 (.73-.80) for Total Affordances in the home. Considering that the quoted alphas for each dimension ranged from 0.66 to 0.76, with 3 out of the 4 dimensions with an alpha >0.7, the questionnaire can be considered as having satisfactory internal validity. Interpretability of the scores was based on quartile analysis and creation of four descriptive categories (Less than Adequate, Moderately Adequate, Adequate, and Excellent) that represent the quality and quantity of home affordances for motor development and its respective dimensions.

Steps in the revision confirmed that the AHEMD-IS is a valid and reliable instrument for the assessment of infants 3 to 18 months of age. The instrument now consists of 35 items divided into 4 dimensions (Physical Space, Variety of Stimulation, Fine-Motor Toys, and Gross-Motor Toys) and a Total score that can be categorized into 4 descriptions of home motor affordances (scoring system): Less than adequate, Moderately Adequate, Adequate, and Excellent. The results suggest that this tool can be a useful instrument for measuring the quantity and quality of affordances in the home environment that are conducive to infant motor development. Researchers can utilize this instrument to account for this very important contributor to infant motor development, while physical therapists can use it for assessment and recommendations for intervention, along with advising parents on home activities.

For a detailed description of the latest development and validation of the AHEMD-IS, readers can visit the journal website^1^. The English version of the questionnaire and score sheet is also published in the article in the appendix. The questionnaire and score sheet versions in Brazilian Portuguese are included as an appendix of this communication and are available for download^9^. There were few modifications in the Brazilian version, e.g., there are no longer references to ethnic background, the educational system has been adjusted to Brazilian parameters, and further observations regarding the risks of using baby walkers and the safety of stairs were added. The final instrument is provided in Appendix 1. Appendix 2 provides the separate score sheets for infants aged 3 to 11 months and infants aged 12 to 18 months, with examples of how to use the AHEMD-IS to improve the home environment. More information about the instrument can also be found on the same website^9^, in both English and Portuguese. We believe that the AHEMD-IS is an important instrument to account for the home as one of the environmental factors influencing infant motor development, and it has noteworthy research^10^ and clinical promise. That promise includes insight to the influence of home motor affordances not only on motor development, but also on future cognitive and social behavior. The Portuguese version of the instrument presented here is the official translation of the latest version of the AHEMD-IS. Dr. Caçola and Dr. Santos would appreciate hearing about data collected using the AHEMD-IS.

## Appendix 1 Affordances no Ambiente Domiciliar para o Desenvolvimento Motor - Escala Bebê (AHEMD-IS) Inventário (3-18 meses)^a^

a^©^ Esse questionário foi desenvolvido pelo Developmental Motor Cognition Lab – University of Texas at Arlington (USA), Motor Development Lab – Texas A&M University (USA) e Laboratório de Pesquisa em Desenvolvimento Neuromotor - Universidade Metodista de Piracicaba (Brasil). Todos os direitos reservados.

## Appendix 2 AFFORDANCES NO AMBIENTE DOMICILIAR PARA O DESENVOLVIMENTO MOTOR ESCALA BEBÊ (AHEMD-IS)^a^

a^©^ Esse questionário foi desenvolvido pelo Developmental Motor Cognition Lab – University of Texas at Arlington (USA), Motor Development Lab – Texas A&M University (USA) e Laboratório de Pesquisa em Desenvolvimento Neuromotor - Universidade Metodista de Piracicaba (Brasil). Todos os direitos reservados.
